# Supplementary material for: Is Hyperuricemia, an Early-Onset Metabolic Disorder, Causally Associated with Cardiovascular Disease Events in Han Chinese?
Source: J Clin Med. 2019 Aug 12;8(8):1202. doi: 10.3390/jcm8081202 (PMC6723695; doi:10.3390/jcm8081202)
Supplement: Supplementary file 1 [file jcm-08-01202-s001.pdf]

Is hyperuricemia, an early-onset metabolic disorder, causally associated with cardiovascular disease events in Han Chinese?

SUPPLEMENTARY MATERIALS

Materials and Methods

Statistical models for the onset sequence study:

In the CVDFACTS, the ages at onset of any CM diseases were left truncated at entry ages in the recruited disease-free participants [1]. For a participant to maintain disease-free status until the final follow-up examination, we recorded the age at onset as right-censored data at the final examination age, whereas for a participant who changed from disease-free to diseased between two consecutive examinations, the age at onset was not accurately observed and was recorded as interval-censored data with these two examination ages [1]. To estimate the sex-specific age-at-onset distributions of each CM disease with the left-truncated and interval-censored data, we used the Turnbull–Frydman estimator [2,3] to plot the overall event time distribution and the conditional distribution of ages at onset of the susceptible subjects as step-function curves. The tail probabilities of overall event curves less than 1 indicated that they were a certain proportion of the nonsusceptible patients for the corresponding diseases [1,4].

Because the right-censored subjects either had later onsets of a disease or were eventually nonsusceptible to the disease, we employed the logistic-GGD location-scale mixture regression models for the left-truncated and interval-censored data [4,5] to simultaneously estimate the sex-specific probability of susceptibility and the age-at-onset distribution of susceptible

subjects for each disease. Model selection [4] was based on the minimum Akaike information criterion together with visual examinations of the closeness between the Turnbull–Frydman empirical event curves [2,3] and the estimated event curves of the fitted regression model [4] (refer to the previous CVDFACTS township-sex-specific analysis of the five CM diseases [1], which provides the model formulation; interpretations of the coefficients of the logistic, location, and scale regression parts in the mixture model [4]; and comparisons of the estimated overall probability density curves across the strata to identify the onset sequences of the associated diseases).

Because this study centered on genetic investigation, we focused only on the sex-specific effects on the CM diseases. The sex-specific distributions of the samples from the CVDFACTS are shown in Table S1, along with the summaries of entry age and censoring status for each disease. According to the Turnbull–Frydman estimator, the estimated probabilities of susceptibility to hyperuricemia ranged from 0.95 to 0.99 until the age of 90 years on the event curves in both sexes (Figure 1(a)). Comparably, the estimated probabilities of susceptibility were 0.98 to 1.00 for hypertension, 0.96 to 1.00 for abdominal obesity, and 0.93 for hypoalphalipoproteinemia in the men. In other words, the participants would eventually develop these diseases, and only the ages at onset differed. Therefore, we fitted the GGD

location-scale regression models without considering susceptibility for these CM diseases and for the left-truncation and interval-censoring data Regarding diabetes mellitus and hypertriglyceridemia in both the men and women and hypo-alpha-lipoproteinemia in the women, some participants were nonsusceptible. We thus fitted two-component models for these diseases by adding a logistic regression part corresponding to the probability of susceptibility.

The optimally fitted models for these six diseases are shown in Table S2. Except for diabetes mellitus, of which the susceptibility fraction and the age at onset were unaltered by sex, sex significantly affected the ages at onset of hyperuricemia, hypertriglyceridemia, and abdominal obesity, as shown by the results from both the location and scale regression parts. A negative location coefficient for the men (−0.83, 95% CI: [−1.21, −0.46]) revealed that the men tended to have earlier ages at onset than did the women for hyperuricemia and hypertriglyceridemia (−0.61, 95% CI: [−0.79, 0.43]). The effect of the location part was observed in the opposite direction for abdominal obesity and with a relatively small magnitude (0.10, 95% CI: [−0.04, 0.24]). Furthermore, a positive scale coefficient showed that the men had a larger variation in ages at onset for hyperuricemia (0.67, 95% CI: [0.38, 0.96]), hypertriglyceridemia (0.51, 95% CI: [0.26, 0.75]), abdominal obesity (0.25, 95% CI: [0.07, 0.42]), hypertension (0.27, 95% CI: [0.13, 0.40]), and hypoalphalipoproteinemia (0.84, 95% CI: [0.24, 1.43]) than had the women.

For disease nonsusceptibility, we found no sex differences regarding the susceptibility fractions of diabetes mellitus (0.50) and hypertriglyceridemia (0.81). However, a sex-based difference was observed for hypoalphalipoproteinemia susceptibility. Although the men had

full susceptibility to hypoalphalipoproteinemia, the estimated probability of the susceptibility of the women was 0.70. Chronologically, the estimated median ages at onset for the susceptible men were 26.1, 31.4, 39.6, 42.4, 58.1, and 61.1 years, and 42.1, 45.5, 61.0, 42.1, 59.8, and 61.1 years for the susceptible women, corresponding to hyperuricemia, hypoalphalipoproteinemia, hypertriglyceridemia, abdominal obesity, hypertension, and diabetes mellitus, respectively.

Self-reported disease status validation

Because the TWB is not currently linked to the National Health Insurance (NHI) database, we used another data set, the CVDFACTS [6], to determine the validity of the self-reported CVD status of community-dwelling Taiwanese. We checked whether the medical costs had been claimed in the NHI system for each reported CVD event. Among the 180 participants who self-reported as CHD patients or stroke patients in the CVDFACTS, 90% were registered in the NHI system with the following International Classification of Diseases, Ninth Revision, Clinical Modification codes: 410-414, 430-438, A290, A291, A292, A293, A294, A299, A270, or A279.

Discussion

Gene functions of the eight identified UA-related genes

The SUA-SNP discovery may broaden our knowledge on how hyperuricemia increases CVD risk. The SNPs located on Chr 4 implicate the influence of the *SLC2A9*, *WDR1*, *IBSP*, *MEPE*, *PKD2*, and *ABCG2* genes on SUA. These genes have been identified as SUA-related genes in both European and Chinese populations [7-10]. *SLC2A9* (solute carrier family 2 member 9) is a transporter for UA and fructose [11]. *ABCG2* (ATP-binding cassette transporter G2) is a known urate exporter [12]. *WDR1* (WD repeat domain 1) [13] may be associated with UA via the mitogen-activated protein kinase signaling pathway [14].

The protein encoded by *IBSP* (integrin binding sialoprotein) is a major structural protein of the bone matrix. Monosodium urate crystals interact with this gene to reduce osteoblast viability, differentiation, and function [15]. *MEPE* (matrix extracellular phosphoglycoprotein) contains a conserved C-terminal region known as the ASARM-motif, which plays a major role in regulating bone mass and cancellous structure. Animal studies have suggested that the N-terminal part of *MEPE* influences purine metabolism and UA production [9].

A recent meta-analysis of 14 GWA studies on SUA suggested that the *PKD2* (polycystic kidney disease 2) gene may be part of a causal pathway determining UA levels in the European

population [16]. *PKD2* encodes the polycystin-2 protein, a multipass membrane protein that functions as a calcium-permeable cation channel, which is involved in calcium transport and calcium signaling in renal epithelial cells. *PKD2* and *SLC2A9* are members of the ion trans-membrane transporter activity pathway (gene ontology: 0015075).

MUC1 (mucin 1, Cell Surface Associated) is a membrane-anchored mucoprotein expressed in the skin, breasts, lungs, gastrointestinal tract, salivary glands, and renal distal tubular cells. *MUC1* is associated with medullary cystic kidney disease type 1 (*MCKD1*). Declining kidney function in people with *MCKD1* leads to hyperuricemia because of impaired UA excretion efficiency [17]. The *GCKR* (glucokinase regulator) gene encodes a regulator of glucokinase, which is a glycolytic enzyme responsible for glucose phosphorylation in the liver. This gene has been associated with SUA in many genome-wide association studies [8] in the European population. Although this gene function is involved in glucose metabolism, a study showed that the association of this gene with SUA is independent of plasma glucose [18].

Figure S1. Flow chart of UA-SNP discovery and tag-SNP selection.

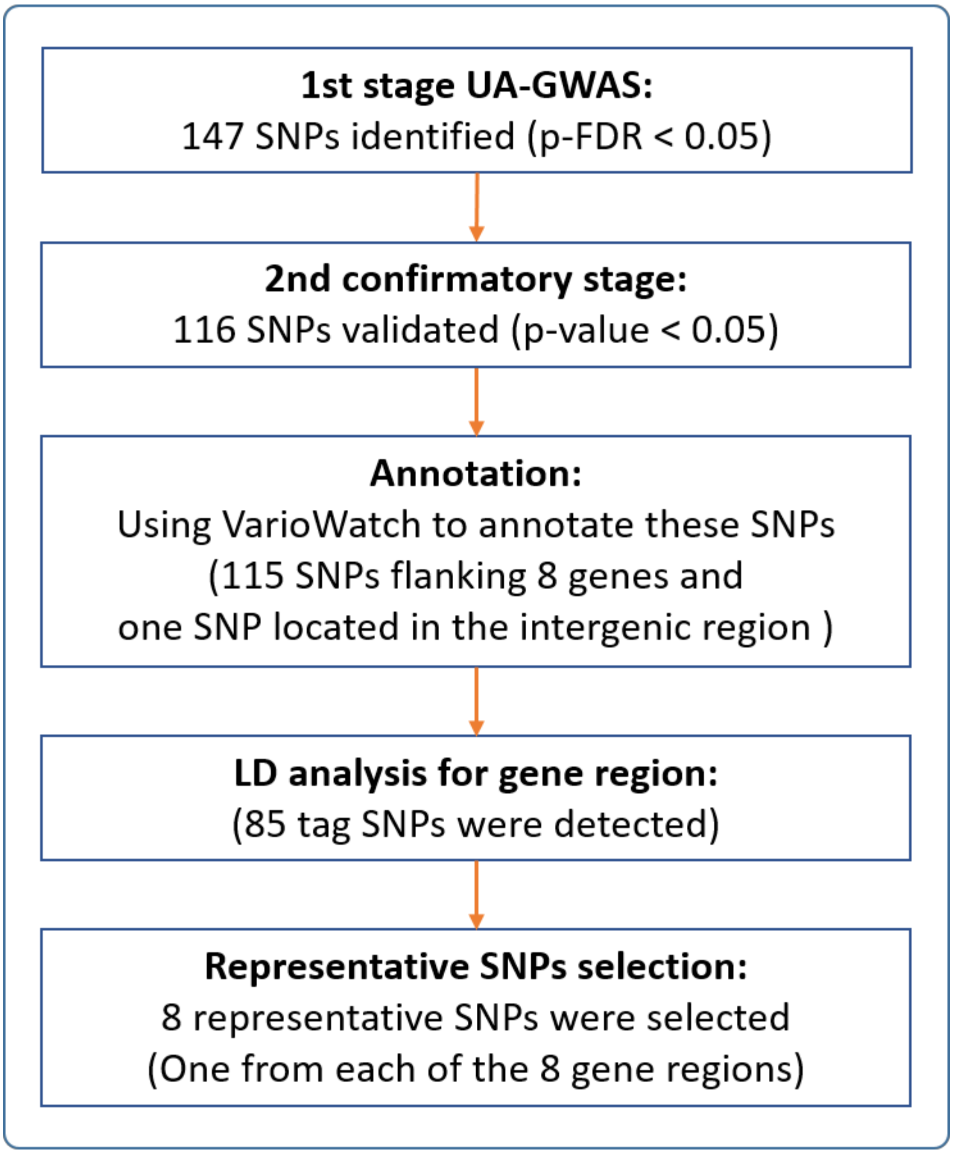

Figure S2. LD plot of the eight identified UA-related genes. (a) *MEPE*, (b) *IBSP*, (c) *GCKR*, (d) *PKD2*, (e) *WDR1*, (f) *ABCG2*, and (g) *SLC2A9*. The most significantly associated SNP is in the red box.

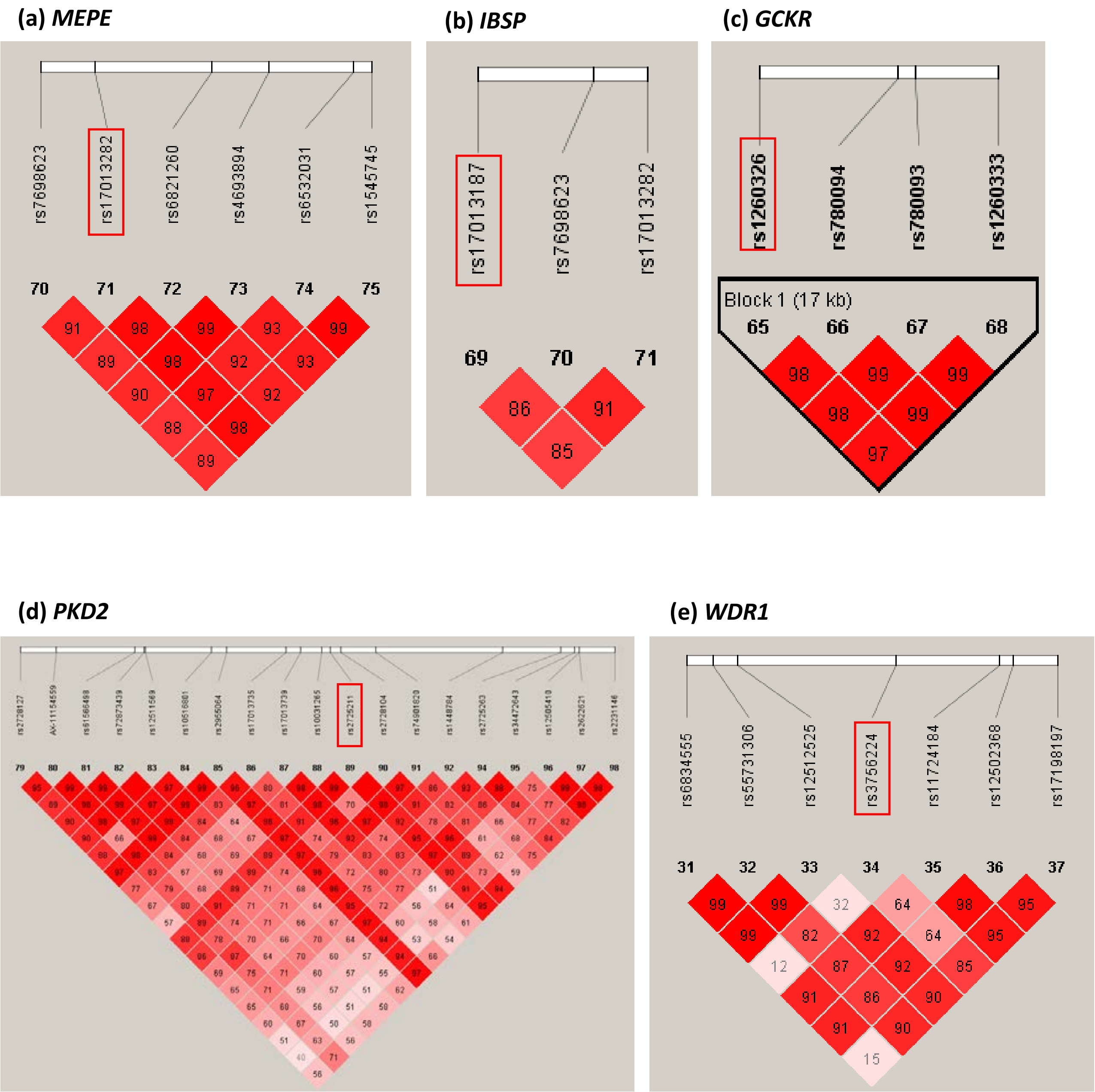

(f) *ABCG2*

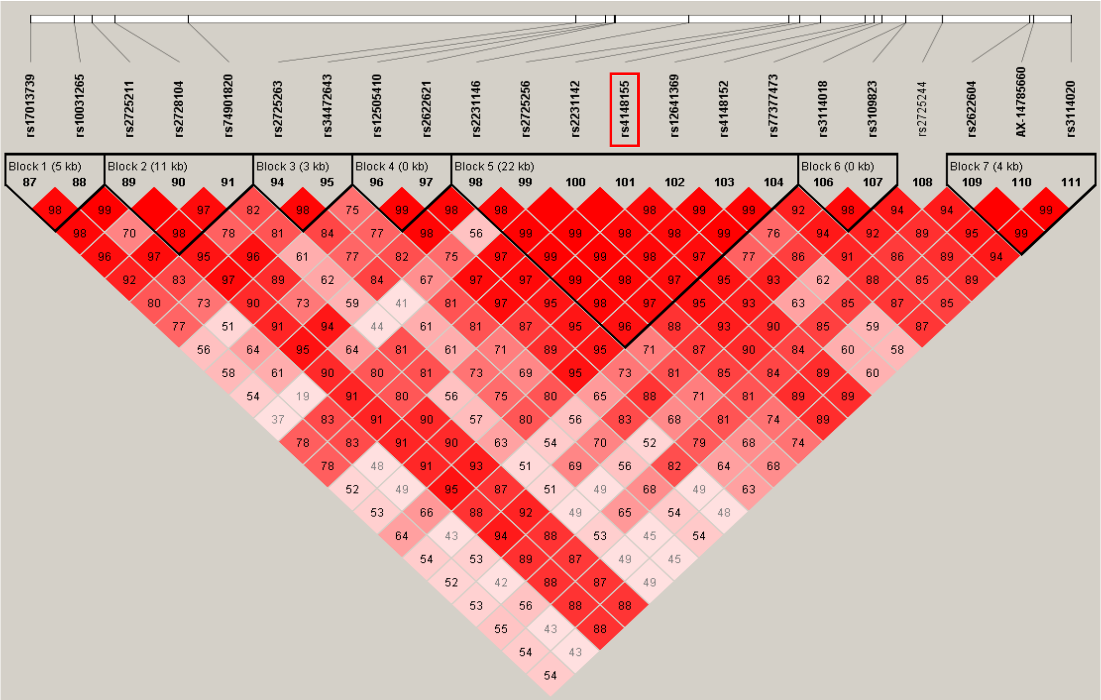

(g) *SLC2A9*

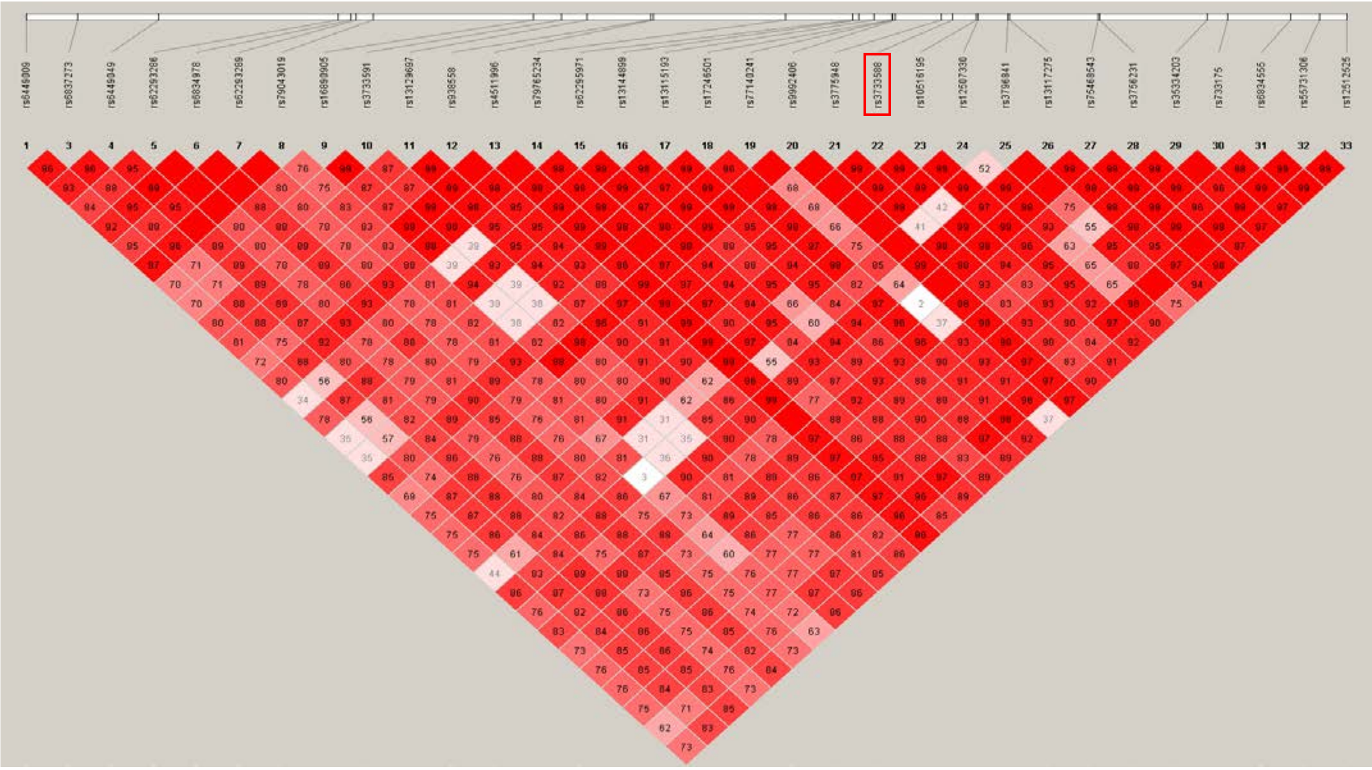

Figure S3. Physical position of the eight representative SNPs.

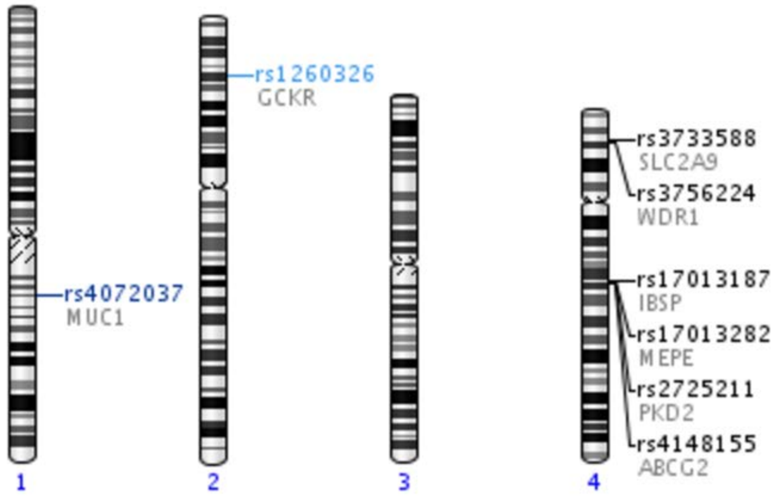

Figure S4. Scatterplot of the genetic associations and causal estimates using the 85 tag-SNPs.

Genetic association with outcome (CVD); genetic association with exposure (SUA).

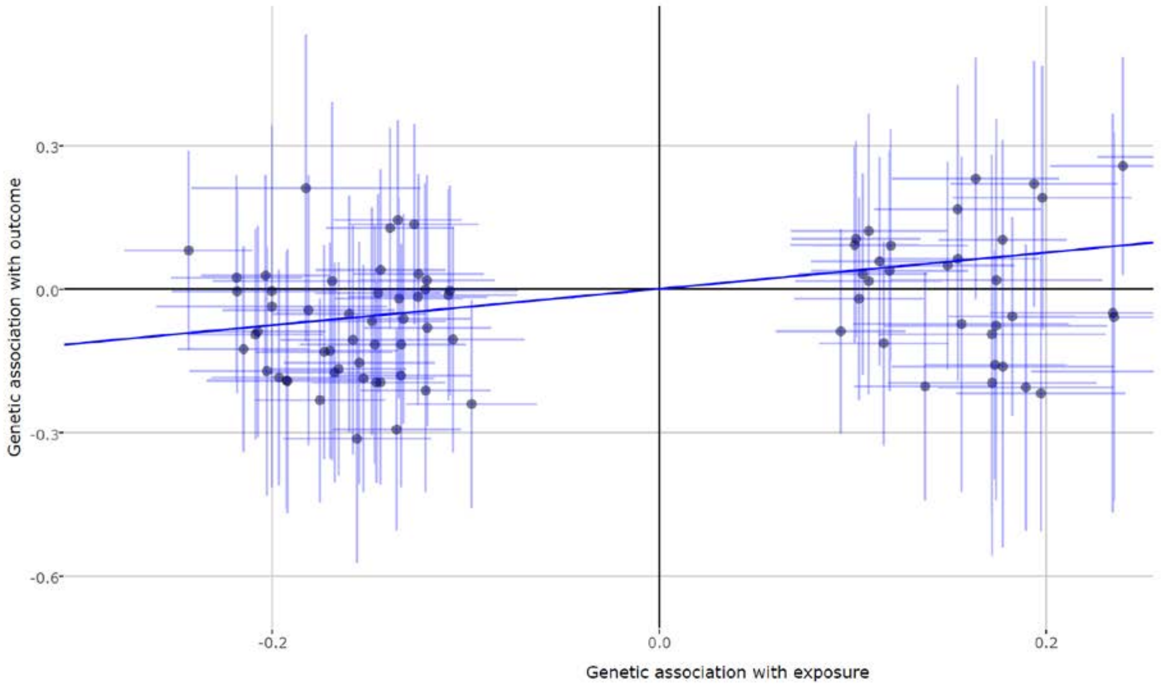

Figure S5. Time to event curves and probability density curves. Estimated event time curves (left panel) and probability density curves (right panel) for (a) CVD, (b) CHD, and (c) stroke stratified by sex and the two-group SUA-WGRS.

(a)

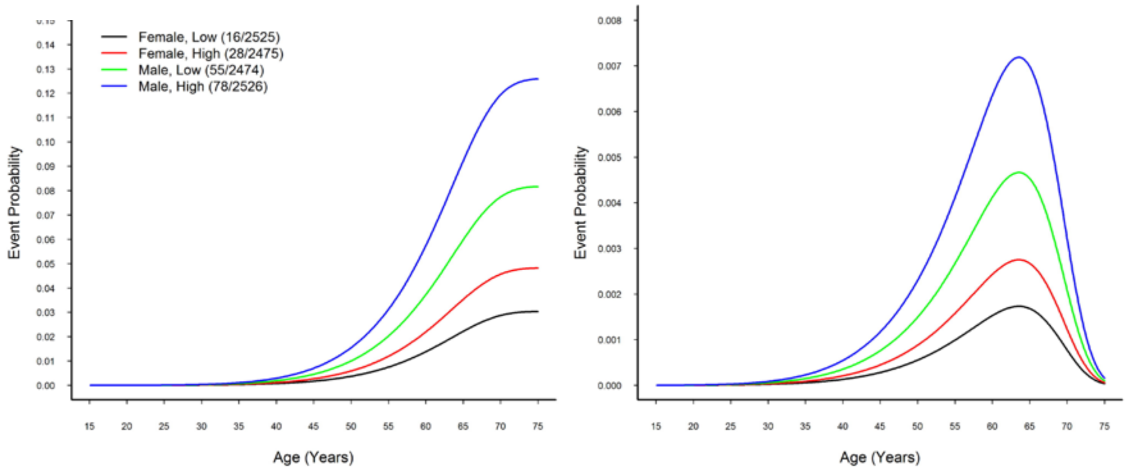

(b)

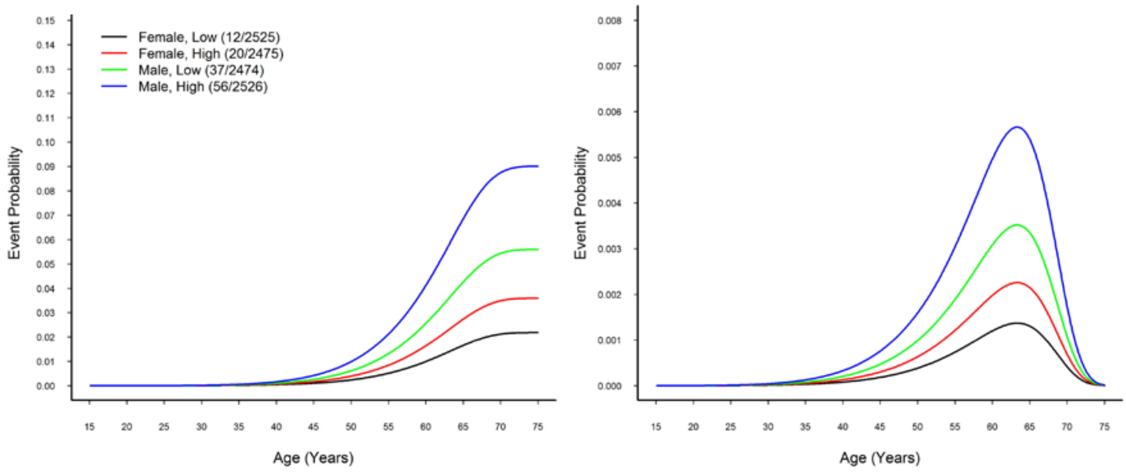

(c)

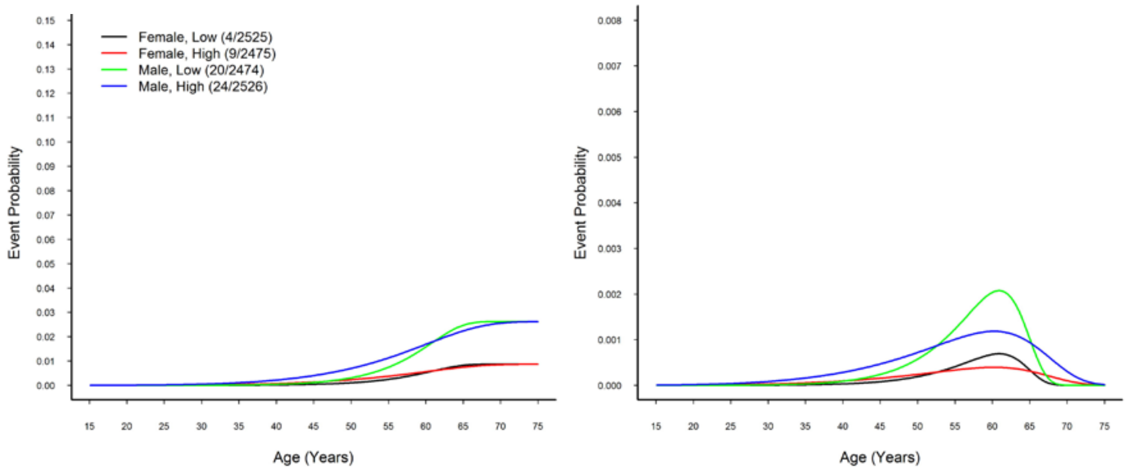

Table S1. Sex-specific counts and percentages of censored observations with medians and interquartile ranges of the age at study entry for hyperuricemia and five CMDs, the CVDFACTS 1989–2002.

Sex	Entry Ages	Censored Data					
	**Median (IQR), years**	**Interval, no.**	**%[a]**	**Right, no.**	**%[a]**	**Total, no.**	**%[b]**
	Diabetes Mellitus						
Female	45.9 (21.5)	159	7	1971	93	2130	56
Male	49.5 (23.7)	136	8	1550	92	1686	44
Total	47.5 (22.3)	295	8	3521	92	3816	
	Hypertension						
Female	42.2 (20.8)	441	19	1830	81	2271	57
Male	45.4 (23.3)	361	21	1338	79	1699	43
Total	43.4 (22.1)	802	20	3168	80	3970	
	Hypertriglyceridemia						
Female	45.4 (22.1)	304	12	2170	88	2474	57
Male	49.5 (24.6)	317	17	1539	83	1856	43
Total	47.1 (23.1)	621	14	3709	86	4330	
	Abdominal Obesity						
Female	40.2 (20.2)	633	33	1273	67	1906	53
Male	47.1 (24.8)	445	27	1229	73	1674	47
Total	42.9 (22.6)	1078	30	2502	70	3580	
	Hypo-α-Lipoproteinemia						
Female	45.2 (21.3)	209	13	1437	87	1646	63
Male	50.6 (24.8)	223	23	759	77	982	37
Total	47.0 (22.7)	432	16	2196	84	2628	
	Hyperuricemia						
Female	44.8 (22.1)	631	27	1738	73	2369	59
Male	49.4 (24.1)	529	32	1118	68	1647	41
Total	46.5 (22.7)	1160	29	2856	71	4016	

CMDs: cardiometabolic diseases, IQR: interquartile range.

[a] Expressed as the row percentage.

[b] Expressed as the column percentage.

Table S2. Optimally fitted logistic-GGD location-scale mixture regression models for hyperuricemia and five CMDs, the CVDFACTS 1989–2002.

Predictors	Logistic		Location		Scale		Shape		AIC
	EST	95% CI	EST	95% CI	EST	95% CI	EST	95% CI	
***(a)* Diabetes Mellitus**									
Intercept	-0.02	-0.33, 0.29	3.90[a]	3.81, 4.00	-1.36[a]	-1.92, -0.81	1.75[c]	0.40, 3.10	2194.10
***(b)* Hypertension**									
Intercept	NA[d]	NA[d]	3.82[a]	3.78, 3.87	-1.14[a]	-1.25, -1.02	1.16[a]	0.87, 1.45	4698.93
Male					0.27[a]	0.13, 0.40			
***(c)* Hypertriglyceridemia**									
Intercept	1.44[a]	1.05, 1.83	3.91[a]	3.81, 4.01	-0.79[a]	-1.04, -0.53	1.16[a]	0.59, 1.73	4276.04
Male			-0.61[a]	-0.79, -0.43	0.51[a]	0.26, 0.75			
***(d)* Abdominal Obesity**									
Intercept	NA[d]	NA[d]	3.41[a]	3.31, 3.51	-0.54[a]	-0.65, -0.44	1.38[a]	0.99, 1.76	5568.76
Male			0.10	-0.04, 0.24	0.25[b]	0.07, 0.42			
***(e)* Hypo-α-Lipoproteinemia**									
Intercept	0.84[a,e]	0.50, 1.18	3.86[a]	3.65, 4.06	-1.06[a]	-1.66, -0.45	3.39[b]	1.11, 5.67	2882.25
Male					0.84[b]	0.24, 1.43			
***(f)* Hyperuricemia**									
Intercept	NA[d]	NA[d]	3.57[a]	3.45, 3.70	-0.39[a]	-0.53, -0.26	1.70[a]	1.18, 2.23	6455.53
Male			-0.83[a]	-1.21, -0.46	0.67[a]	0.38, 0.96			

AIC: Akaike information criterion, CI: confidence interval, CMD: cardiometabolic disease, EST: estimated regression coefficient, NA: not available.

[a] $P < 0.001$, [b] $P < 0.05$, [c] $P < 0.1$ for 2-sided P values of the Wald test.

[d] 1-component model for both sex strata.

[e] 1-component model for the male subjects and 2-component model for the female subjects.

Table S3. Characteristics of the participants (continuous traits).

	CVD case (N=179)	Non-CVD (N=9821)	
	Mean (SD)	Mean (SD)	p-value
Age (year)	58.7 (7.3)	48.7 (11.1)	<0.0001
BMI (Kg/M^2)	25.8 (3.3)	24.3 (3.6)	<0.0001
Uric acid (mg/dl)	6.2 (1.5)	5.7 (1.5)	<0.0001
Fasting Glucose (mg/dl)	108.6 (28.4)	96.4 (21.5)	<0.0001
Total Cholesterol (mg/dl)	182.6 (37.3)	193.0 (35.4)	<0.0001
TG (mg/dl)	132.1 (83.5)	118.3 (98.3)	0.03
HDL-C (mg/dl)	46.7 (11.7)	53.3 (13.1)	<0.0001
LDL-C (mg/dl)	114.5 (33.7)	120.7 (31.5)	0.009
eGFR ($mL/min/1.73m^2$)	89.7 (25.2)	103.0 (24.7)	<0.0001
SBP (mmHg)	127.2 (16.9)	116.1 (17.3)	<0.0001
DBP (mmHg)	75.3 (10.8)	72.2 (11.1)	0.0002

BMI: body mass index, TG: triglyceride, HDL-C: high-density lipoprotein cholesterol, LDL-C: low-density lipoprotein cholesterol, SBP: systolic blood pressure, DBP: diastolic blood pressure. eGFR ($mL/min/1.73m^2$) = $175 \times (Scr)^{-1.154} \times (Age)^{-0.203} \times$ (0.742 if a woman).

Table S3. Characteristics of participants (categorical traits).

	CVD case (N=179)	Non-CVD (N=9821)	
	N (%)	N (%)	P value
**Sex**			
Male	133 (74.3)	4867 (49.6)	<0.0001
Female	46 (25.7)	4954 (50.4)	
**Drinking habit**			
Yes	15 (8.4)	783 (7.9)	<0.0001
No	143 (79.9)	8737 (89.0)	
Quit	21 (11.7)	301 (3.1)	
**Smoking habit**			
Yes	23 (12.9)	1136 (11.6)	<0.0001
Few	21 (11.7)	819 (8.3)	
No	84 (46.9)	6746 (68.7)	
Quit	51 (28.5)	1120 (11.4)	
**Education**			
Elementary School	33 (18.4)	678 (6.9)	<0.0001
Junior-high/Senior-high	76 (42.5)	4043 (41.2)	
BS/MS/PhD	70 (39.1)	5094 (51.9)	
**Marriage**			
Single	4 (2.2)	1147 (11.7)	0.0002
Married	150 (83.8)	7699 (78.5)	
Divorce/Widowed	25 (14.0)	967 (9.9)	
**Regular Exercise**			<0.0001
No	78 (43.6)	5801 (59.1)	
Yes	101 (56.4)	4020 (40.9)	

Table S4. Sample characteristics by WGRS quartile (Male).

Characteristic	wGRS (Male)				p-value†
	Q1(N=1237)	Q2(N=1237)	Q3(N=1304)	Q4(N=1222)	
	Mean (SD)	Mean (SD)	Mean (SD)	Mean (SD)	
**SUA (mg/dl)**	6.1 (1.2)	6.4 (1.4)	6.6 (1.4)	6.8 (1.5)	***<0.0001****
**Age (yr)**	49.5 (11.5)	49.5 (11.4)	49.0 (11.3)	49.2 (11.1)	*0.630*
**BMI (kg/m²)**	25.0 (3.4)	25.2 (3.5)	25.2 (3.4)	25.1 (3.4)	*0.605*
**FG (mg/dl)**	100.1 (25.8)	99.8 (25.2)	99.3 (21.4)	101.1 (27.6)	*0.338*
**T-CHO (mg/dl)**	191.3 (36.4)	190.6 (33.5)	189.9 (32.7)	193.2 (37.8)	*0.107*
**TG (mg/dl)**	134.7 (125.6)	130.5 (94.3)	132.4 (87.0)	145.0 (146.4)	***0.010****
**HDL-C (mg/dl)**	48.8 (11.4)	48.3 (10.7)	48.2 (11.1)	47.8 (11.1)	*0.199*
**LDL-C (mg/dl)**	121.2 (32.8)	122.5 (31.0)	121.2 (30.1)	122.9 (30.8)	*0.392*
**eGFR (mL/min/1.73m²)**	95.6 (20.1)	93.9 (20.0)	94.1 (20.1)	94.4 (20.5)	*0.147*
**SGOT (U/L)**	26.5 (21.5)	26.3 (13.4)	26.7 (15.6)	26.0 (11.0)	*0.680*
**SGPT (U/L)**	29.3 (22.1)	30.0 (24.2)	30.8 (28.3)	29.3 (22.2)	*0.351*
**SBP (mmHg)**	120.1 (15.8)	120.4 (16.1)	120.6 (16.0)	120.9 (16.7)	*0.650*
**DBP (mmHg)**	75.4 (10.2)	75.9 (10.3)	76.2 (10.9)	76.7 (11.0)	***0.030****

SUA: serum uric acid; **BMI**: body mass index; **FG**: fasting glucose; **HDL-C/LDL-C**: high-/low-density lipoprotein cholesterol; **SBP/DBP**: systolic/diastolic blood pressure. *: $P < 0.05$. †: Chi-square test for categorical variables. Analysis of variance for continuous variables. Variables with skewed distribution were log-transformed before input for analysis. eGFR (mL/min/1.73 m²) = 175 × $(Scr)^{-1.154}$ × $(Age)^{-0.203}$ × (0.742 if women) × (1.212 if African-American).

0

Table S4. Sample characteristics by WGRS quartile (Female).

Characteristic	wGRS (Female)				p-value†
	Q1(N=1253)	Q2(N=1271)	Q3(N=1195)	Q4(N=1280)	
	Mean (SD)	Mean (SD)	Mean (SD)	Mean (SD)	
**SUA (mg/dl)**	4.6 (1.0)	4.8 (1.1)	5.0 (1.1)	5.2 (1.2)	***<0.0001****
**Age (yr)**	48.8 (11.2)	48.3 (10.7)	48.1 (10.6)	48.4 (11.0)	*0.434*
**BMI (kg/m²)**	23.4 (3.6)	23.5 (3.6)	23.4 (3.7)	23.5 (3.6)	*0.924*
**FG (mg/dl)**	93.1 (15.6)	92.3 (14.1)	93.3 (15.7)	93.9 (21.7)	*0.127*
**T-CHO (mg/dl)**	195.4 (35.4)	193.0 (36.5)	194.2 (35.4)	195.0 (35.5)	*0.342*
**TG (mg/dl)**	101.5 (73.5)	97.5 (66.3)	102.1 (72.7)	105.3 (78.3)	*0.061*
**HDL-C (mg/dl)**	58.6 (13.4)	58.3 (13.5)	57.9 (12.7)	57.6 (13.1)	*0.269*
**LDL-C (mg/dl)**	119.3 (30.9)	118.3 (33.1)	119.5 (32.1)	120.0 (31.3)	*0.592*
**eGFR (mL/min/1.73m²)**	111.0 (25.7)	111.4 (25.6)	111.0 (26.3)	111.0 (26.5)	*0.966*
**SGOT (U/L)**	22.6 (9.3)	22.8 (11.3)	22.5 (9.3)	22.5 (9.4)	*0.827*
**SGPT (U/L)**	19.7 (13.1)	20.6 (17.5)	20.4 (15.0)	20.4 (15.1)	*0.444*
**SBP (mmHg)**	111.5 (17.0)	111.6 (16.9)	112.4 (17.7)	113.1 (18.4)	*0.080*
**DBP (mmHg)**	67.8 (9.9)	68.3 (10.1)	68.8 (10.5)	69.1 (10.4)	***0.080****

SUA: serum uric acid; **BMI**: body mass index; **FG**: fasting glucose; **HDL-C/LDL-C**: high-/low-density lipoprotein cholesterol; **SBP/DBP**: systolic/diastolic blood pressure. *: $P < 0.05$. †: Chi-square test for categorical variables. Analysis of variance for continuous variables. Variables with skewed distribution were log-transformed before input for analysis. eGFR (mL/min/1.73 m²) = 175 × $(Scr)^{-1.154}$ × $(Age)^{-0.203}$ × (0.742 if women) × (1.212 if African-American).

Table S5. Information on the four satisfactorily replicated Han Chinese–specific SUA-SNPs.

			European			Han-Chinese			Our Study		
**Gene**	**SNP**	**Chr**	**MAF**	**Beta**	***p*-value**	**MAF**	**Beta**	***p*-value**	**MAF**	**Beta**	***p*-value**
TRIM46	rs11264341	1	0.43(T)	-0.05	6.2×10^{-19}	0.33(C)	0.017	4.49×10^{-3}	0.29(C)	0.086	5.74×10^{-6}
GCKR	rs780094	2	0.42(T)	0.05	1.4×10^{-9}	0.44(T)	0.013	0.014	0.48(T)	0.101	2.58×10^{-9}
SLC2A9	rs11722228	4	0.50(T)	0.167	1.75×10^{-75}	0.31(T)	0.028	3.68×10^{-6}	0.42(G)	-0.2433	1.73×10^{-46}
ABCG2	rs4148155	4	0.11(A)	-0.17	1.5×10^{-25}	0.29(A)	-0.046	9.23×10^{-15}	0.31(G)	0.3116	4.34×10^{-66}

Chr.: chromosome, MAF: minor allele frequency.

Table S6. Fitted logistic-GGD location-scale mixture regression model with sex and the two-group SUA-WGRS$_L$ for CVD.

Predictors	Logistic		Location		Scale		Shape		AIC
	OR (95% CI)	p-value	EST (95% CI)	p-value	EST (95% CI)	p-value	EST (95% CI)	p-value	
Intercept	1	Referent	4.16[a] (4.11,4.21)	<0.001	-2.37[a] (-2.99,-1.74)	<0.001	1.43[b] (0.37,2.49)	0.008	2593.094
Male	2.84[a] (1.99,4.06)	<0.001							
High	1.47[c] (1.07,2.02)	0.018							

AIC: Akaike information criterion, **CI**: confidence interval, **EST**: regression coefficient, **OR**: odds ratio, **WGRS$_L$**: weighted genetic risk score from the literature.

[a] $P < 0.001$, [b] $P < 0.01$, and [c] $P < 0.05$ for 2-sided P values of the Wald test.

Table S7. Results of the MR-Egger regressions.

	85 tag-SNPs				8 representative SNPs			
	Estimate	SE	95% CI	p-value	Estimate	SE	95% CI	p-value
Intercept	0.006	0.05	(-0.09,0.11)	0.90	0.050	0.11	(-0.16,0.27)	0.63
MR-Egger	0.34	0.31	(-0.26,0.95)	0.27	0.32	0.54	(-0.73,1.37)	0.55

The intercept from the MR-Egger regression provided a test for directional pleiotropy.

Table S8. Fitted logistic-GGD location-scale mixture regression models with sex and the two-group SUA-WGRS for CVD, CHD, and stroke.

Predictors	Logistic		Location		Scale		Shape		AIC
	OR (95% CI)	p-value	EST (95% CI)	p-value	EST (95% CI)	p-value	EST (95% CI)	p-value	
***(a)* CVD (n=179)**									
Intercept	1	Referent	4.16[a] (4.11,4.21)	<0.001	-2.37[a] (-3.00,-1.73)	<0.001	1.42[b] (0.35,2.50)	0.009	2589.89
Male	2.85[a] (1.99,4.07)	<0.001							
High	1.62[b] (1.17,2.23)	0.003							
***(b)* CHD (n=125)**									
Intercept	1	Referent	4.16[a] (4.11,4.20)	<0.001	-2.47[a] (-3.39,-1.55)	<0.001	1.45 (-0.17,3.08)	0.079	1896.382
Male	2.66[a] (1.75,4.03)	<0.001							
High	1.67[b] (1.15,2.43)	0.007							
***(c)* Stroke (n=57)**									
Intercept	1	Referent	4.11[a] (4.05,4.18)	<0.001	-2.68[a] (-3.44,-1.93)	<0.001	1.56[c] (0.31,2.82)	0.015	993.513
Male	3.07[a] (1.64,5.73)	<0.001							
High					0.57[c] (0.08,1.06)	0.023			

AIC: Akaike information criterion, **CI**: confidence interval, **EST**: estimated regression coefficient, **OR**: estimated odds ratio, **WGRS**: weighted genetic risk score, n: number of cases.

[a] $P < 0.001$, [b] $P < 0.01$, and [c] $P < 0.05$ for 2-sided P values of the Wald test.

Table S9. Fitted logistic-GGD location-scale mixture regression model with sex and the two-group HDL-WGRS for CVD.

Predictors	Logistic		Location		Scale		Shape		AIC
	OR (95% CI)	p-value	EST (95% CI)	p-value	EST (95% CI)	p-value	EST (95% CI)	p-value	
Intercept	1	Referent	4.16[a] (4.11,4.22)	<0.001	-2.36[a] (-3.00,-1.72)	<0.001	1.42[c] (0.34,2.49)	0.010	2598.383
Male	2.84[a] (1.99,4.06)	<0.001							
High	0.91 (0.66,1.24)	0.539							

AIC: Akaike information criterion, **CI**: confidence interval, **EST**: estimated regression coefficient, **OR**: estimated odds ratio, **WGRS**: weighted genetic risk score.

[a] $P < 0.001$, [b] $P < 0.01$, and [c] $P < 0.05$ for 2-sided P values of the Wald test.

Table S10. Fitted logistic-GGD location-scale mixture regression model with sex and the two-group new SUA-GRS for CVD.

Predictors	Logistic		Location		Scale		Shape		AIC
	OR (95% CI)	p-value	EST (95% CI)	p-value	EST (95% CI)	p-value	EST (95% CI)	p-value	
Intercept	1	Referent	4.16[a] (4.11,4.21)	<0.001	-2.37[a] (-3.01,-1.73)	<0.001	1.43[c] (0.35,2.51)	0.010	2590.721
Male	2.85[a] (2.00,4.08)	<0.001							
High	1.58[b] (1.15,2.18)	0.005							

AIC: Akaike information criterion, **CI**: confidence interval, **EST**: estimated regression coefficient, **OR**: estimated odds ratio, **WGRS**: weighted genetic risk score.

[a] $P < 0.001$, [b] $P < 0.01$, and [c] $P < 0.05$ for 2-sided P values of the Wald test.

References

1. Tsay, Y.C.; Chen, C.H.; Pan, W.H. Ages at Onset of 5 Cardiometabolic Diseases Adjusting for Nonsusceptibility: Implications for the Pathogenesis of Metabolic Syndrome. *Am J Epidemiol* **2016**, *184*, 366-377, doi:10.1093/aje/kwv449.
2. Frydman, H. A Note on Nonparametric Estimation of the Distribution Function from Interval-Censored and Truncated Observations. *Journal of the Royal Statistical Society. Series B (Methodological)* **1994**, *56*, 71-74.
3. Turnbull, B.W. The Empirical Distribution Function with Arbitrarily Grouped, Censored and Truncated Data. *Journal of the Royal Statistical Society. Series B (Methodological)* **1976**, *38*, 290-295.
4. Chen, C.H.; Tsay, Y.C.; Wu, Y.C.; Horng, C.F. Logistic-AFT location-scale mixture regression models with nonsusceptibility for left-truncated and general interval-censored data. *Stat Med* **2013**, *32*, 4285-4305, doi:10.1002/sim.5845.
5. Tsay Y.C.,Chen C.H., Wu YC, Horng CF. MixtureRegLTIC: Mixture regression models for left-truncated and interval-censored data. R package version 1.0.0. Availabe online: https://CRAN.R-project.org/package=MixtureRegLTIC (accessed on
6. Chuang, S.Y.; Bai, C.H.; Chen, W.H.; Lien, L.M.; Pan, W.H. Fibrinogen independently predicts the development of ischemic stroke in a Taiwanese population: CVDFACTS study. *Stroke* **2009**, *40*, 1578-1584, doi:10.1161/STROKEAHA.108.540492.
7. Yang, B.; Mo, Z.; Wu, C.; Yang, H.; Yang, X.; He, Y.; Gui, L.; Zhou, L.; Guo, H.; Zhang, X., et al. A genome-wide association study identifies common variants influencing serum uric acid concentrations in a Chinese population. *BMC Med Genomics* **2014**, *7*, 10, doi:10.1186/1755-8794-7-10.
8. Kottgen, A.; Albrecht, E.; Teumer, A.; Vitart, V.; Krumsiek, J.; Hundertmark, C.; Pistis, G.; Ruggiero, D.; O'Seaghdha, C.M.; Haller, T., et al. Genome-wide association analyses identify 18 new loci associated with serum urate concentrations. *Nat Genet* **2013**, *45*, 145-154, doi:10.1038/ng.2500.
9. Zelenchuk, L.V.; Hedge, A.M.; Rowe, P.S. Age dependent regulation of bone-mass and renal function by the MEPE ASARM-motif. *Bone* **2015**, *79*, 131-142, doi:10.1016/j.bone.2015.05.030.
10. Charles, B.A.; Shriner, D.; Doumatey, A.; Chen, G.; Zhou, J.; Huang, H.; Herbert, A.; Gerry, N.P.; Christman, M.F.; Adeyemo, A., et al. A genome-wide association study of serum uric acid in African Americans. *BMC Med Genomics* **2011**, *4*, 17, doi:10.1186/1755-8794-4-17.
11. Vitart, V.; Rudan, I.; Hayward, C.; Gray, N.K.; Floyd, J.; Palmer, C.N.; Knott, S.A.; Kolcic, I.; Polasek, O.; Graessler, J., et al. SLC2A9 is a newly identified urate transporter influencing serum urate concentration, urate excretion and gout. *Nat Genet* **2008**, *40*, 437-442, doi:10.1038/ng.106.
12. Takada, T.; Ichida, K.; Matsuo, H.; Nakayama, A.; Murakami, K.; Yamanashi, Y.; Kasuga, H.; Shinomiya, N.; Suzuki, H. ABCG2 dysfunction increases serum uric acid by decreased intestinal urate excretion. *Nucleosides Nucleotides Nucleic Acids* **2014**, *33*, 275-281, doi:10.1080/15257770.2013.854902.
13. Liu, L.J.; Zhang, X.Y.; He, N.; Liu, K.; Shi, X.G.; Feng, T.; Geng, T.T.; Yuan, D.Y.; Kang, L.L.; Jin, T.B. Genetic variation in WDR1 is associated with gout risk and gout-related metabolic indices in the Han Chinese population. *Genet Mol Res* **2016**, *15*, doi:10.4238/gmr.15027381.

14. Yao Zhang, K.L., Lifeng Ma, Kangping Liu, Xugang Shi, Yuan Zhang, Na He, Yiduo Zhao, Xikai Zhu, Tianbo Jin, Longli Kang. Associations of gout with polymorphisms in SLC2A9, WDR1, CLNK, PKD2, and ABCG2 in Chinese Han and Tibetan populations. *Int J Clin Exp Pathol* **2016**, *9*, 66-67-7762.
15. Chhana, A.; Callon, K.E.; Pool, B.; Naot, D.; Watson, M.; Gamble, G.D.; McQueen, F.M.; Cornish, J.; Dalbeth, N. Monosodium urate monohydrate crystals inhibit osteoblast viability and function: implications for development of bone erosion in gout. *Ann Rheum Dis* **2011**, *70*, 1684-1691, doi:10.1136/ard.2010.144774.
16. Lee, Y.H.; Song, G.G. Pathway analysis of genome-wide association studies on uric acid concentrations. *Hum Immunol* **2012**, *73*, 805-810, doi:10.1016/j.humimm.2012.05.004.
17. Bleyer, A.J.; Kmoch, S.; Antignac, C.; Robins, V.; Kidd, K.; Kelsoe, J.R.; Hladik, G.; Klemmer, P.; Knohl, S.J.; Scheinman, S.J., et al. Variable clinical presentation of an MUC1 mutation causing medullary cystic kidney disease type 1. *Clin J Am Soc Nephrol* **2014**, *9*, 527-535, doi:10.2215/CJN.06380613.
18. Sun, X.; Jiang, F.; Zhang, R.; Tang, S.S.; Chen, M.; Peng, D.F.; Yan, J.; Wang, T.; Wang, S.Y.; Bao, Y.Q., et al. Serum uric acid levels are associated with polymorphisms in the SLC2A9, SF1, and GCKR genes in a Chinese population. *Acta Pharmacol Sin* **2014**, *35*, 1421-1427, doi:10.1038/aps.2014.87.
